# A major genetic component of BSE susceptibility

**DOI:** 10.1186/1741-7007-4-33

**Published:** 2006-10-02

**Authors:** Katrin Juling, Hermann Schwarzenbacher, John L Williams, Ruedi Fries

**Affiliations:** 1 Chair of Animal Breeding, Technical University of Munich, Hochfeldweg 1, 85354 Freising-Weihenstephan, Germany; 2 Division of Genetics and Genomics, Roslin Institute, Roslin, Midlothian EH25 9PS, UK; 3 CERSA, Parco Tecnologico Padano, Via Einstein 26900 Lodi, Italy

## Abstract

**Background:**

Coding variants of the prion protein gene (*PRNP*) have been shown to be major determinants for the susceptibility to transmitted prion diseases in humans, mice and sheep. However, to date, the effects of polymorphisms in the coding and regulatory regions of bovine *PRNP *on bovine spongiform encephalopathy (BSE) susceptibility have been considered marginal or non-existent. Here we analysed two insertion/deletion (indel) polymorphisms in the regulatory region of bovine *PRNP *in BSE affected animals and controls of four independent cattle populations from UK and Germany.

**Results:**

In the present report, we show that two previously reported 23- and 12-bp insertion/deletion (indel) polymorphisms in the regulatory region of bovine *PRNP *are strongly associated with BSE incidence in cattle. Genotyping of BSE-affected and control animals of UK Holstein, German Holstein, German Brown and German Fleckvieh breeds revealed a significant overrepresentation of the deletion alleles at both polymorphic sites in diseased animals (P = 2.01 × 10^-3 ^and P = 8.66 × 10^-5^, respectively). The main effect on susceptibility is associated with the 12-bp indel polymorphism. Compared with non-carriers, heterozygous and homozygous carriers of the 12-bp deletion allele possess relatively higher risks of having BSE, ranging from 1.32 to 4.01 and 1.74 to 3.65 in the different breeds. These values correspond to population attributable risks ranging from 35% to 53%.

**Conclusion:**

Our results demonstrate a substantial genetic *PRNP* associated component for BSE susceptibility in cattle. Although the BSE risk conferred by the deletion allele of the 12-bp indel in the regulatory region of *PRNP* is substantial, the main risk factor for BSE in cattle is environmental, i.e. exposure to feedstuffs contaminated with the infectious agent.

## Background

Endogenous prion protein is known to play a central role in the pathogenesis of transmitted prion diseases [1,2]. The gene encoding the prion protein, *PRNP*, has therefore been suggested as a candidate locus for disease susceptibility. In humans, mice and sheep, coding variants of *PRNP *have been shown to have major effects on the susceptibility to and incubation time of such diseases [3-8]. However, no clear relationships between bovine *PRNP *polymorphisms and susceptibility to bovine spongiform encephalopathy (BSE) have been revealed so far [9-12]. Here we examine the effects of two insertion/deletion (indel) polymorphisms that were tentatively associated with BSE in a small case-control study [11]. Both the 23-bp indel [11] in the promoter region and the 12-bp indel [13] in intron 1 affect binding sites for transcription factors (RP58 and SP1, respectively), and thus might affect the expression of *PRNP *[14]. We analysed both polymorphisms in BSE-affected and control animals of four different populations: Holstein-Friesian cattle from the United Kingdom (UK Holstein) and Germany (German Holstein), German Brown and German Fleckvieh.

## Results

### Single marker analyses

**Figure 1:**
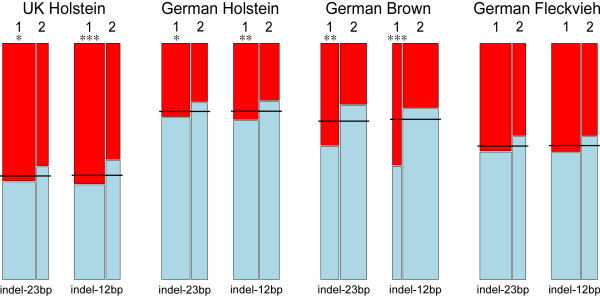
**Allele Frequencies**. Allele frequencies of the 23- and 12-bp insertion/deletion polymorphisms in UK Holstein, German Holstein, German Brown and German Fleckvieh BSE affected (red) and control (light-blue) animals. "1" and "2" above the columns represent the deletion and insertion alleles, respectively. The horizontal black lines indicate the expected proportions of cases and controls in each class. The thickness of the columns is proportional to the allele frequency. Asterisks indicate the level of significance: * < 0.05, ** < 0.01, *** < 0.001.

Genotyping of both indel polymorphisms and χ^2 ^testing revealed significant association of each polymorphism with the BSE status in all populations except German Fleckvieh. Genotypes with deletion alleles and deletion alleles of both loci are generally overrepresented in the affected animals of all breeds (Table 1, Table 2 and Figure 1). Using Fisher's combined probability test, highly significant associations between the deletion allele and the BSE cases were found for both the 23-bp (P = 2.01 × 10^-3^) and the 12-bp indel (P = 8.66 × 10^-5^) over all populations. A combined logistic regression analysis involving all breeds also revealed highly significant associations between both indels and BSE (P = 5.7 × 10^-5 ^and P = 1.2 × 10^-7^, respectively). Modelling the genotype data shows that the BSE risk tends to increase in line with the number of deletion alleles at both loci (Table 2). Heterozygous and homozygous carriers of the 12-bp deletion allele, when compared with non-carriers, have relatively higher risks of BSE, ranging from 1.32 to 4.01 and 1.74 to 3.65 in the different breeds. The corresponding population attributable risks [15,16], i.e. the portion of the BSE incidence in the population that is due to the risk allele (12-bp deletion allele), range from to 0.35 to 0.53 (Table 2).

### Haplotype analyses

**Figure 2:**
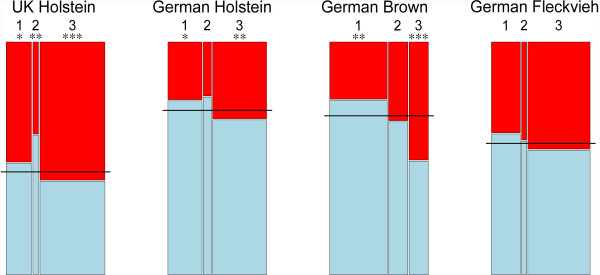
**Haplotype Frequencies**. Haplotype frequencies in UK Holstein, German Holstein, German Brown and German Fleckvieh BSE affected (red) and control (light blue) animals. Numbers above the columns represent the haplotypes: (1) 23ins-12ins, (2) 23del-12ins and (3) 23del-12del. The horizontal black lines indicate the expected proportions of cases and controls in each class. The thickness of the columns is proportional to the haplotype frequency. Stars indicate the level of significance: * < 0.05, ** < 0.01, *** < 0.001.

We performed haplotype analysis, to estimate the combined effect of both polymorphisms. Strong linkage disequilibrium was present in all populations (e.g. in UK Holstein: D' = 0.99 C.I. = 0.96 – 1.0, LOD = 145.31; r^2 ^= 0.745), with the most frequent haplotypes being 23del-12del, 23ins-12ins and 23del-12ins. Haplotype 23ins-12del occurs in < 1% in all populations and was therefore excluded from further analyses. The χ^2 ^test indicated significant deviations from the expected haplotype frequencies in cases and controls of all populations except German Fleckvieh (Figure 2 and Table 3) and logistic regression modelling revealed significant contrasts in the ORs. The 23del-12del haplotype is associated with an increased risk of BSE incidence, whereas the 23ins-12ins haplotype seems to be protective. The effect of the 23del-12ins haplotype depends on the population, but it does not differ significantly from that of the 23ins-12ins reference haplotype in any population. Additionally, the effect of the 23del-12del haplotype in the combined analysis is highly significantly different from that of the remaining major haplotypes (P = 3.7 × 10^-6 ^and P = 9.6 × 10^-4 ^for the 23ins-12ins and 23del-12ins haplotypes, respectively).

### Diplotype analyses

**Figure 3:**
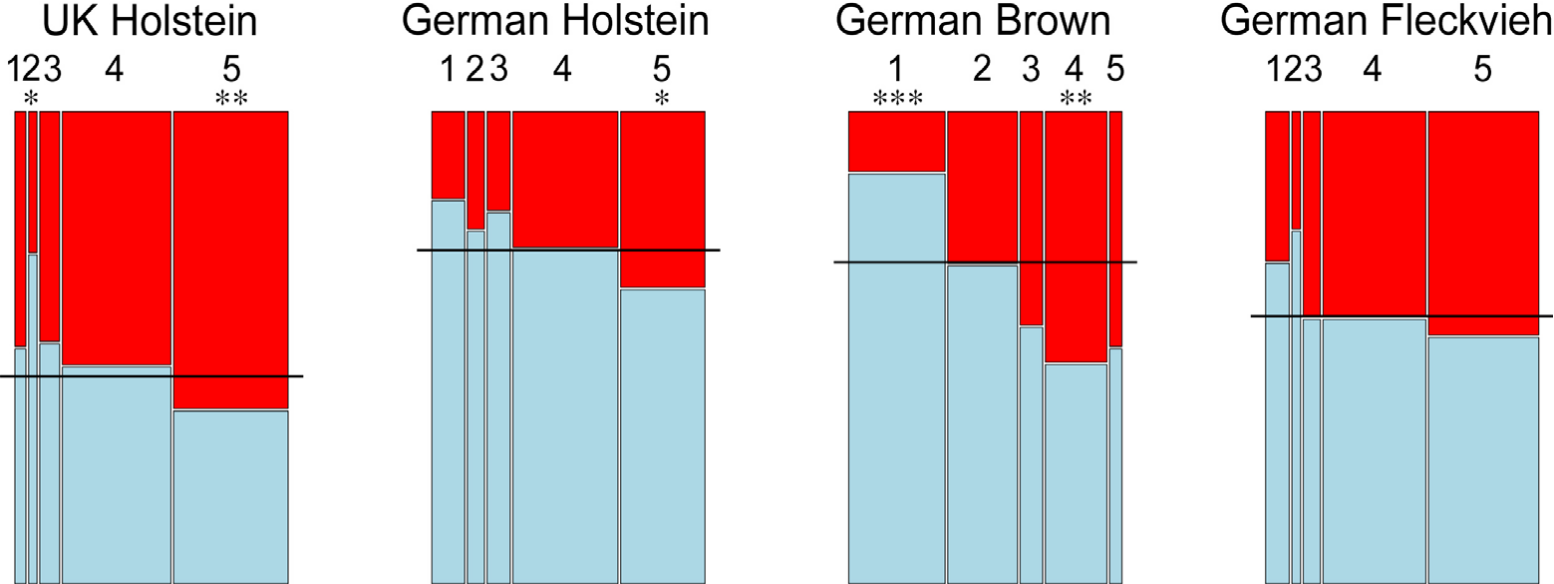
**Diplotype Frequencies**. Diplotype frequencies in UK Holstein, German Holstein, German Brown and German Fleckvieh BSE affected (red) and control (light blue) animals. Numbers above the columns represent the haplotypes: (1) 23ins-12ins/23ins-12ins, (2) 23ins-12ins/23del-12ins, (3) 23del-12ins/23del-12del, (4) 23ins-12ins/23del-12del and (5) 23del-12del/23del-12del. The horizontal black lines indicate the expected proportions of cases and controls in each class. The thickness of the columns is proportional to the diplotype frequency. Asterisks indicate the level of significance: * < 0.05, ** < 0.01, *** < 0.001.

**Figure 4:**
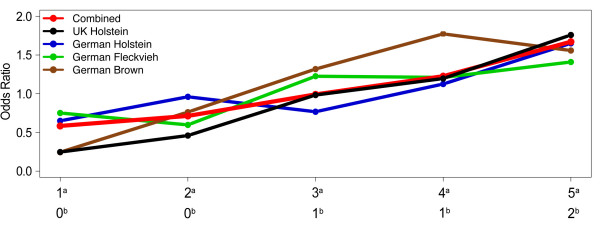
**Odds Ratios of Diplotypes**. Odds ratios (ORs) conferred by the diplotypes in each population and over all populations. The ORs were calculated using the absolute risks averaged across all five diplotypes as reference. ^a^Numbers refer to diplotypes: (1) 23ins-12ins/23ins-12ins, (2) 23ins-12ins/23del-12ins, (3) 23del-12ins/23del-12del, (4) 23ins-12ins/23del-12del and (5) 23del-12del/23del-12del. ^b^Numbers indicate number of deletion alleles at the 12-bp indel locus.

We attempted to assess the risk of BSE infection associated with each diplotype. Five common diplotypes were found (Figure 3 and Table 4), with the sixth, 23del-12ins/23del-12ins, being relatively rare (< 3 %); as such, it was excluded from further analyses. For German Holstein control animals, the diplotypes were inferred from estimated haplotype frequencies (see Additional File 1: Inferring allele, genotype, haplotype and diplotype frequencies from half-sibs). In both Holstein populations, the 23del-12del/23del-12del diplotype is significantly overrepresented in affected animals as revealed by χ^2 ^testing (P = 2.5 × 10^-3 ^and P = 9.2 × 10^-3 ^for UK and German Holstein, respectively). Significant association was also observed in the German Brown breed, where the 23ins-12ins/23ins-12ins diplotype is highly overrepresented in the controls (P = 4.7 × 10^-4^), and the 23del-12del/23ins-12ins diplotype occurs significantly more often in the cases (P = 6.0 × 10^-4^). Analysis over all populations indicates that the highest risk is conferred by the 23del-12del/23del-12del diplotype (Table 4). Figure 4 shows the diplotype conferred ORs obtained from logistic regression analysis of the combined data, accounting for population effects, and the data of each individual population. The ORs were calculated using the absolute risks averaged across all five diplotypes as reference in order to allow for a meaningful comparison of populations. The analyses of the individual populations and the combined analysis suggest that risk of BSE in a population tends to increase in line with the number of deletion alleles at the 12-bp indel.

### Modelling the modes of allelic action and relative contribution of both polymorphisms

Likelihood ratio tests were applied as proposed by North et al. [17] to investigate both the modes of allelic action for both loci (additive versus dominance effects) and for possible epistatic effects (additive by additive, additive by dominance and dominance by dominance interactions). The results indicate that for the individual populations as well as for the combined analysis neither the inclusion of dominance effects nor inclusion of epistatic effects leads to a significantly better fit compared to a model that accounts for additive effects only. Hence, likelihood ratio tests were performed to verify whether fitting an additive effect for either indel locus reduces the likelihood significantly compared to a full model that includes additive effects of both polymorphisms. This analysis revealed that exclusion of the 23-bp indel does not reduce the likelihood significantly in any of the individual populations or in the combined data. However, exclusion of the 12-bp indel polymorphism reduces the likelihood significantly in UK Holstein (P = 1.04 × 10^-3^) and in the combined analysis (P = 6.89 × 10^-4^). Thus, the main effect on BSE susceptibility seems to result from the 12-bp indel. This is supported by the haplotype analysis, which showed that the effect of the 23del-12ins haplotype does not differ significantly from that of 23ins-12ins, whereas the 23del-12del haplotype is significantly associated with higher BSE risk in the Holstein populations and in German Brown (Table 3).

## Discussion

We found significant association between *PRNP *promoter indel polymorphisms and the BSE status in three cattle populations. The association is not significant in the German Fleckvieh population. However, as in the other populations, the deletion alleles are overrepresented in BSE-affected animals of German Fleckvieh. Furthermore, including the German Fleckvieh population into the combined analyses leads to more significant overall association (data not shown). Thus, there is no reason to assume a fundamentally different situation in German Fleckvieh with regard to the effect of the indel polymorphisms on BSE susceptibility. Haplotype analysis and likelihood ratio tests identified that the main effect on BSE susceptibility is due to the 12-bp indel. Although suggestive, our findings cannot directly verify a causal role for either or both indel polymorphisms in the aetiology of BSE. The observed associations could result from another, genetic variant in linkage disequilibrium with the indel polymorphisms, although a functional role for these two polymorphisms is supported by their location in regulatory regions of *PRNP*. *In vitro *analyses have shown that the 12-bp deletion disrupts binding of the SP1 transcription factor and *in vivo *and *in vitro *investigations suggest that the two polymorphisms affect *PRNP *expression, albeit with the direction of the effects remaining to be clarified [14]. One could speculate that, all other factors being equal, susceptibility to BSE might be reduced with a lowered expression of endogenous prion protein. Elevated expression of the prion protein, in contrast, would result in more substrate for conversion into the pathogenic form and possibly higher BSE susceptibility.

The calculated population attributable risks allow assessing the effect of the indel polymorphisms on the BSE epidemic. For example, 53% and 35% of the Holstein BSE cases in UK and Germany, respectively, can be explained by the 12-bp deletion allele (Table 2). Thus, if the UK Holstein population had been fixed for the alternative 12-bp insertion allele (and a possible effect on time to onset is not considered), roughly 80,000 BSE cases would have occurred instead of the 184,000 cases that were recorded. Similarly, the number of cases in German Holstein would have been reduced from 138 to about 90. Although the two populations have similar frequencies of the risk increasing deletion alleles, the BSE-incidence is orders of magnitude smaller in the German than in the UK population, which is most likely explained by differences in environmental exposure, such as a generally less intensive use of meat and bone meal in ruminant nutrition in Germany. Thus, although the indel polymorphisms – and particularly the 12-bp indel – are associated with the BSE risk, they are not the main risk factors. Instead, the decisive factor is environmental: that is to say, whether or not an animal is sufficiently exposed to the infectious agent. Eliminating or minimising exposure to the infectious agent should therefore be considered to be the primary measure to reduce BSE incidence, even in the light of the strong genetic component of BSE susceptibility as we report it here. However, selective breeding to reduce the frequency of the susceptibility alleles would still bring an additional protective component that should not be discounted.

## Conclusion

In this study we presented significant association between *PRNP *promoter indel polymorphisms and the BSE status in three of four investigated cattle populations (Figure 1, Table 1 and Table 2). We conclude that the main effect on BSE susceptibility seems to result from the 12-bp indel. The causality of either or both polymorphism cannot be verified in this study, however our data highlight that the 12 bp deletion allele is associated with increased risk for an animal to succumb to feed-borne BSE (Table 4 and Figure 4). Thus, our findings show a substantial genetic component for the susceptibility of cattle to BSE, conferred by variants in the regulatory region of *PRNP*, a gene that has been shown to be involved in prion disease susceptibility/resistance in other species. However, the different dimensions of the BSE epidemic in UK and Germany, on the one hand, and the similar frequencies of the *PRNP*-associated susceptibility alleles in UK and German cattle, on the other, indicate, that the main BSE risk factor for cattle is environmental, i.e. exposure to contaminated feed, and not genetic.

## Methods

### Subjects and controls

The BSE status of German animals was diagnosed in clinically-suspect animals and by testing all slaughtered animals 24 months of age or older in certified local laboratories based on the presence of proteinase K resistant prion protein fragments. The initial diagnosis had to be confirmed by the German National BSE Reference Laboratory at the Friedrich-Löffler Institute (Riems Island, Germany). If suitable tissue was available, the diagnosis was histopathologically confirmed. DNA from German BSE affected animals was extracted by the Friedrich-Löffler Institute using the QiAamp DNA Mini Kit (Qiagen Valencia, CA, USA) after tissue decontamination with 13.5 M guanidine chloride. A total of 276 cases collected from November 2000 until the end of 2005 were available. Based on the farmer's declaration, 127 of these cases were of German Holstein, 106 were of German Fleckvieh and 43 were of the German Brown breed. Control animals for the German Holstein case group consisted of 627 paternal half-sibs that were approximately contemporaneous with the BSE animals and had geographically similarly distributions. Only the maternally-inherited alleles were considered (see Additional File 1: Inferring allele, genotype, haplotype and diplotype frequencies from half-sibs). Control animals (n = 90) for German Brown cases were selected from bulls used for artificial insemination such that their pedigree was representative for the German Brown population. The German Fleckvieh control group (n = 137) consisted of bulls kept at the experimental Station Hirschau of the Technical University of Munich. All were purchased at markets throughout Bavaria and can be considered to be representative for the German Fleckvieh population. Samples of BSE-affected UK animals were collected between 1990 and 1993. Animals were identified initially as BSE suspects by veterinary diagnosis of live animals, with the initial diagnosis being confirmed by histopathological examination following culling. Blood samples were obtained from a total of 365 BSE affected and 276 BSE unaffected age-matched paternal half-sib offspring that were born on the same farms in the same birth cohorts as the BSE-affected animals. The proportion of affected to unaffected animals was similar in each of the 37 half-sib groups. None of the controls were subsequently identified as BSE cases in the database of the UK Department for Environment, Food and Rural Affairs (Defra). DNA was obtained from blood via proteinase K digestion and phenol-extraction.

### Genotyping

Sequence information was obtained from GenBank accession number AJ298878. UK and German Holstein animals were genotyped by single base extension reactions (iPLEX™ Assay, SEQUENOM™, San Diego, CA, USA), with the first base of the insertion and the first base after the insertion representing the insertion and deletion alleles, respectively. Alleles were detected by MALDI-TOF mass spectrometry (MassARRAY^®^, SEQUENOM™, San Diego, CA, USA). German Brown and German Fleckvieh case and control animals were genotyped using PCR, followed by high resolution agarose (3%) gel electrophoresis to visualise the allelic PCR products. PCR primer 5'-CCTGTTGAGCGTGCTCGT-3' and 5'-ACCTGCGGCTCCTCTACC-3' were used for genotyping the 23-bp indel (191bp/168bp) and primer 5'-GGAAGTCACGTGAAGGCACT-3' and 5'-CAAAGAGTTGGACAGGCACA-3' for the 12-bp indel (215bp/203bp).

### Statistical analyses

Statistical analyses were performed using the R software environment [18], extended by the packages genetics (version 1.2.0) and logistf (version 1.05) [19]. Haploview 3.2 [20] based on the expectation maximisation (EM) method was used to infer linkage disequilibrium and Phase 2.1.1 [21,22], which applies a Bayesian statistical framework, was used to derive haplotypes and individual diplotypes. Diplotypes with posterior probabilities lower than 0.9 were excluded from further analysis. For the calculation of population attributable risks, population genotype frequencies were inferred by weighting frequency estimates obtained for cases and based on the average annual incidence rate of BSE (UK: 0.2 %, Germany: 0.0012 % [23]).

## Authors' contributions

KJ conceived the study and participated in its design, set up DNA samples, carried out the genotyping, performed the data analysis, participated in the statistical analysis and prepared the manuscript. HS performed statistical analyses and participated in study design and helped to draft the manuscript. JLW organised the collection of the UK samples, participated in the study design and helped to draft the manuscript. RF coordinated the project and participated in the study design and in drafting the manuscript. All authors read and approved the final manuscript.

## Supplementary Material

Additional file 1 **Inferring allele, genotype, haplotype and diplotype frequencies from half-sibs**. Supplementary methods: Control animals for the German Holstein case group consisted of paternal half-sibs. Details on inferring the maternally inherited alleles. Click here for file

## Figures and Tables

**Table 1:** Summary of genotype association analyses with BSE status

	**23-bp indel**					**12-bp indel**				
	Frequency of Genotypes					Frequency of Genotypes				
				
**UK Holstein**	Cases^a^	(n)	Controls^a^	(n)	P-Value^c^	Cases^a^	(n)	Controls^a^	(n)	P-Value^c^
del/del	55.4	(201)	46.4	(128)	2.4 × 10^-2^	49.4	(173)	37.0	(100)	2.1 × 10^-3^
del/ins	41.0	(149)	48.9	(135)	4.7 × 10^-2^	45.4	(159)	51.9	(140)	1.1 × 10^-1^
ins/ins	3.6	(13)	4.7	(13)	4.7 × 10^-1^	5.1	(18)	11.1	(30)	5.8 × 10^-3^
**German Holstein^b^**										

del/del	45.7	(58)	38.0^b^	(119)^b^	1.4 × 10^-1^	40.0	(50)	28.2^b^	(87)^b^	1.6 × 10^-2^
del/ins	46.5	(59)	47.3^b^	(148)^b^	8.8 × 10^-1^	45.6	(57)	49.8^b^	(154)^b^	4.2 × 10^-1^
ins/ins	7.9	(10)	14.7^b^	(46)^b^	5.2 × 10^-2^	14.4	(18)	22.0^b^	(68)^b^	7.2 × 10^-2^
**German Brown**										

del/del	20.9	(9)	13.8	(12)	2.9 × 10^-1^	7.0	(3)	3.3	(3)	3.4 × 10^-1^
del/ins	65.1	(28)	41.4	(36)	1.1 × 10^-2^	51.2	(22)	22.2	(20)	7.8 × 10^-4^
ins/ins	14.0	(6)	44.8	(39)	5.0 × 10^-4^	41.9	(18)	74.4	(67)	2.5 × 10^-4^
**German Fleckvieh**										

del/del	53.8	(57)	46.3	(63)	2.5 × 10^-1^	45.3	(48)	39.4	(54)	3.6 × 10^-1^
del/ins	39.6	(42)	43.4	(59)	5.6 × 10^-1^	46.2	(49)	45.3	(62)	8.8 × 10^-1^
ins/ins	6.6	(7)	10.3	(14)	3.1 × 10^-1^	8.5	(9)	15.3	(21)	1.1 × 10^-1^

^a ^Represents frequency (%) of genotypes; (n) indicates the number of genotypes.

^b ^Control consists of half-sibs (see Additional File 1: Inferring allele, genotype, haplotype and diplotype frequencies from half-sibs).

^c ^P-values of χ^2^-test against the both remaining genotypes

**Table 2:** Summary of single marker association analyses with BSE status

	Frequency of Deletion				P-Value^c^	Logistic Regression Analysis			
			
**23-bp indel**	Cases^a^	(n)	Controls^a^	(n)		P-Value^d^	OR ins/del^e^	OR del/del^e^	PAR^f^
UK Holstein	75.9	(363)	70.8	(276)	4.2 × 10^-2^	2.9 × 10^-2^	1.36	1.84	0.24
German Holstein	68.9	(127)	61.8^b^	(335)	4.3 × 10^-2^	4.4 × 10^-2^	1.38	1.91	0.46
German Brown	53.5	(43)	34.5	(87)	3.3 × 10^-3^	5.4 × 10^-3^	4.74	4.62	0.69
German Fleckvieh	73.6	(106)	68.0	(136)	1.8 × 10^-1^	1.9 × 10^-1^	1.31	1.71	0.36
Combined		(639)		(834)	2.0 × 10^-3 g^	5.7 × 10^-5^	1.43	2.04	
**12-bp indel**									

UK Holstein	72.1	(350)	63.0	(270)	5.8 × 10^-4^	3.2 × 10^-4^	1.61	2.60	0.53
German Holstein	62.8	(125)	53.2^b^	(357)	9.1 × 10^-3^	1.1 × 10^-2^	1.48	2.20	0.35
German Brown	32.6	(43)	14.4	(90)	5.9 × 10^-4^	1.1 × 10^-2^	4.01	3.65	0.44
German Fleckvieh	68.4	(106)	62.0	(137)	1.5 × 10^-1^	1.5 × 10^-1^	1.32	1.74	0.44
Combined		(624)		(854)	8.7 × 10^-5 g^	1.2 × 10^-7^	1.58	2.50	

^a ^Represents frequency (%) of deletion allele as calculated from genotypes; (n) indicates the number of individuals.

^b ^Control consists of half-sibs (see Additional File 1: Inferring allele, genotype, haplotype and diplotype frequencies from half-sibs).

^c ^P-values of χ^2^-test.

^d ^P-values from logistic regression, modelling the number of deletion alleles on BSE status.

^e ^Odds Ratios with reference genotype: ins/ins.

^f ^Population Attributable Risk: the portion of the BSE incidence in the population that is due to 12-bp deletion allele [15, 16].

^g ^P-Values of Fisher's Combined Probability test.

**Table 3:** Summary of haplotype association analyses with BSE status

	Frequency of Haplotypes					Logistic Regression Analysis		
			
**UK Holstein**	Cases^a^	(n)	Controls^a^	(n)	P-Value^c^	OR^d^	P-Value 23ins-12ins^e^	P-Value 23del-12ins^f^
23ins-12ins	24.2	(171)	29.1	(159)	5.1 × 10^-2^	1		6.6 × 10^-2^
23del-12ins	4.1	(29)	8.1	(44)	3.1 × 10^-3^	0.62	6.6 × 10^-2^	
23del-12del	71.7	(506)	62.8	(343)	8.9 × 10^-4^	1.37	1.6 × 10^-2^	1.3 × 10^-3^
**German Holstein**								

23ins-12ins	30.2	(77)	39.1^b^	(232)	1.4 × 10^-2^	1		8.1 × 10^-1^
23del-12ins	7.2	(17)	9.1^b^	(56)	3.9 × 10^-1^	0.93	8.1 × 10^-1^	
23del-12del	62.2	(162)	51.3^b^	(327)	7.5 × 10^-3^	1.49	1.4 × 10^-2^	1.1 × 10^-1^
**German Brown**								

23ins-12ins	45.0	(39)	66.1	(119)	1.3 × 10^-3^	1		1.8 × 10^-1^
23del-12ins	22.5	(18)	19.4	(35)	5.7 × 10^-1^	1.58	1.8 × 10^-1^	
23del-12del	32.5	(27)	14.4	(26)	7.8 × 10^-4^	3.14	5.4 × 10^-4^	8.4 × 10^-2^
**German Fleckvieh**								

23ins-12ins	26.8	(58)	32.6	(90)	1.7 × 10^-1^	1		7.5 × 10^-1^
23del-12ins	5.1	(11)	5.4	(15)	8.7 × 10^-1^	1.15	7.5 × 10^-1^	
23del-12del	68.1	(147)	62.0	(171)	1.7 × 10^-1^	1.33	1.5 × 10^-1^	7.2 × 10^-1^
**Combined**								

23ins-12ins						1		5.0 × 10^-1^
23del-12ins						0.90	5.0 × 10^-1^	
23del-12del						1.50	3.7 × 10^-6^	9.6 × 10^-4^

^a ^Represent frequency (%) of haplotypes; (n) indicates the number of haplotypes.

^b ^Control consists of half-sibs (see Additional File 1: Inferring allele, genotype, haplotype and diplotype frequencies from half-sibs).

^c ^P-values from χ^2^-test against pooled observations of all remaining haplotypes.

^d ^Odds Ratios with reference haplotype 23ins-12ins.

^e ^P-values from logistic regression, testing the risk effect compared to reference haplotype 23ins-12ins.

^f ^P-values from logistic regression, testing the risk effect compared to reference haplotype 23del-12ins.

**Table 4:** Summary of diplotype association analyses with BSE status

	No. of 12-bp Deletion	Frequency of Diplotypes					
				
**UK Holstein**		Cases^a^	(n)	Controls^a^	(n)	P-Value^d^	OR^e^
23del-12ins/23del-12ins	0	0	(0)	<1.0	(3)	-	
23ins-12ins/23ins-12ins^c^	0	3.7	(13)	4.8	(13)	4.8 × 10^-1^	1^c^
23del-12ins/23ins-12ins	0	1.7	(6)	5.1	(14)	1.4 × 10^-2^	0.45
23del-12ins/23del-12del	1	6.5	(23)	8.8	(24)	2.7 × 10^-1^	0.96
23ins-12ins/23del-12del	1	39.0	(139)	43.6	(119)	2.4 × 10^-1^	1.17
23del-12del/23del-12del	2	48.7	(172)	36.6	(100)	3.6 × 10^-3^	1.72
**German Holstein**							

23del-12ins/23del-12ins	0	<1.0	(1)	<1.0	(3)^b^	-	
23ins-12ins/23ins-12ins^c^	0	7.8	(10)	15.3	(47)^b^	6.1 × 10^-2^	1^c^
23del-12ins/23ins-12ins	0	5.5	(7)	7.1	(22)^b^	6.0 × 10^-1^	1.48
23del-12ins/23del-12del	1	6.3	(8)	9.4	(29)^b^	2.4 × 10^-1^	1.18
23ins-12ins/23del-12del	1	39.1	(50)	40.6	(125)^b^	3.4 × 10^-2^	1.73
23del-12del/23del-12del	2	40.6	(52)	26.6	(82)^b^	1.2 × 10^-2^	2.54
**German Brown**							

23del-12ins/23del-12ins	0	2.4	(1)	3.3	(3)	-	
23ins-12ins/23ins-12ins^c^	0	14.3	(6)	45.6	(41)	3.7 × 10^-4^	1^c^
23del-12ins/23ins-12ins	0	26.2	(11)	25.6	(23)	9.6 × 10^-1^	3.12
23del-12ins/23del-12del	1	11.9	(5)	6.7	(6)	3.1 × 10^-1^	5.40
23ins-12ins/23del-12del	1	38.1	(16)	15.6	(14)	4.3 × 10^-3^	7.27
23del-12del/23del-12del	2	7.1	(3)	3.3	(3)	-	6.38
**German Fleckvieh**							

23del-12ins/23del-12ins	0	<1.0	(1)	0	(0)	-	
23ins-12ins/23ins-12ins^c^	0	6.5	(7)	10.9	(15)	2.4 × 10^-1^	1^c^
23del-12ins/23ins-12ins	0	1.9	(2)	4.3	(6)	-	0.79
23del-12ins/23del-12del	1	6.5	(7)	6.5	(9)	1	1.63
23ins-12ins/23del-12del	1	38.9	(42)	39.1	(54)	1	1.61
23del-12del/23del-12del	2	45.4	(49)	39.1	(54)	2.9 × 10^-1^	1.88
**Combined**							

23del-12ins/23del-12ins	0						
23ins-12ins/23ins-12ins^c^	0						1^c^
23del-12ins/23ins-12ins	0						1.22
23del-12ins/23del-12del	1						1.70
23ins-12ins/23del-12del	1						2.10
23del-12del/23del-12del	2						2.86

^a ^Represents the frequency (%) of diplotypes; (n) indicates the number of individuals.

^b ^Calculated assuming Hardy-Weinberg equilibrium from haplotype frequency estimates (see Additional File 1: Inferring allele, genotype, haplotype and diplotype frequencies from half-sibs).

^c ^Diplotype taken as reference.

^d ^P-values from χ^2^-test against pooled observations of all remaining diplotypes.

^e^Odds Ratios from logistic regression.
