# Supplementary material for: A major genetic component of BSE susceptibility
Source: BMC Biol. 2006 Oct 2;4:33. doi: 10.1186/1741-7007-4-33 (PMC1601964; doi:10.1186/1741-7007-4-33)
Supplement: Additional file 1 — Inferring allele, genotype, haplotype and diplotype frequencies from half-sibs. Supplementary methods: Control animals for the German Holstein case group consisted of paternal half-sibs. Details on inferring the maternally inherited alleles. [file 1741-7007-4-33-S1.pdf]

A major genetic component of BSE susceptibility

Supplementary Methods

Inferring allele and genotype frequencies from half-sibs

The German Holstein control animals were paternal half-sibs. Thus, the actual control frequencies were those of the maternally inherited alleles. Because the sire was heterozygous at both loci, only progeny with homozygous genotypes could be considered because only these genotypes allowed us to unequivocally determine the origin of each allele (Additional Table 1). Maternal genotype frequencies were estimated based on the inferred maternal allele frequencies, assuming Hardy-Weinberg equilibrium (Additional Table 2).

Additional Table 1: Calculation of allele frequencies in dams of German Holstein half-sibs.

Genotype Sire	Possible Genotypes Daughters[a] n_1	n_2	n_3	Allele Frequencies in Dams[b]	
AB	AA	AB	BB	$p(A) = \frac{n_1}{n_1 + n_3}$	$p(B) = \frac{n_3}{n_1 + n_3}$

[a] n_1, n_2, n_3 indicate the number of half-sibs of genotype AA, AB and BB, respectively.
[b] p(A),p(B) indicate frequency of Allele A and B, respectively.

Additional Table 2: Calculation of the genotype frequencies in dams of German Holstein half-sibs

	Genotype Frequencies in Maternal Population [a,b]		
Genotypes	AA	AB	BB
Formula	$(n_1 + n_3) \cdot p(A)^2$	$(n_1 + n_3) \cdot 2p(A) \cdot p(B)$	$(n_1 + n_3) \cdot p(B)^2$

[a] n_1, n_2, n_3 indicate the number of half-sibs of genotype AA, AB and BB, respectively.
[b] p(A),p(B) indicate frequency of Allele A and B, respectively.

Inferring haplotype and diplotype frequencies from half-sibs

Diplotypes of the half-sib animals were obtained using PHASE 2.1.1 [1, 2]. The sire transmitted either haplotype 23del-12del or 23ins-12ins to progeny (see Additional Table 3). Thus, except for daughters with the diplotype 23del-12del / 23ins-12ins, the maternal haplotype could be directly determined by subtraction of the paternal haplotype. Because of the large number of animals with diplotype 23del-12del / 23ins-12ins, we decided to indirectly calculate the frequency of maternally contributed haplotypes from these diplotypes as shown in Additional Table 4. Diplotype frequencies in the dam population were estimated based on the maternal haplotype frequencies, assuming Hardy-Weinberg equilibrium (Additional Table 5). Haplotype 4 (23ins-12del) occurred with a frequency <1% and therefore was excluded from the estimation of diplotype frequencies in the dams. Calculation of the approximative numbers of diplotypes were based on half the number of half-sibs because each animal contributed only one haplotype observation.

Additional Table 3: Calculation of haplotype frequencies in dams based on observed diplotypes in German Holstein half-sibs.

			Maternal Haplotype			
			**1**	**2**	**3**	**4**
Diplotype	Number of Diplotype Observations		23del-12ins	23del-12del	23ins-12ins	23ins-12del
23del-12ins[a] / 23ins-12ins [b]	$n(1/3)$	40	40			
23del-12ins[a] / 23del-12del [b]	$n(1/2)$	16	16			
23del-12del[a] / 23del-12del [b]	$n(2/2)$	181		181		
**23del-12del / 23ins-12ins [c]**	$n(2/3)$	253		$253 \cdot p(2)_e$ [d] 145.98	$253 \cdot p(3)_e$ [d] 107.02	
23ins-12ins[a] / 23ins-12ins [b]	$n(3/3)$	125			125	
23del-12del [b] / 23ins-12del[a]	$n(2/4)$	3				3
∑	n	618	56	327	232	3
P_D (x)[e]			**0.0906**	**0.5291**	**0.3754**	**0.0049**

[a] maternal haplotype.
[b] paternal haplotype.
[c] origin of haplotypes cannot be directly determined.
[d] $p(2)_e$ and $p(3)_e$ indicate the frequency of maternally inherited haplotype 23del-12del and 23ins-12ins among half-sibs with diplotype 23del-12del / 23ins-12ins, respectively (see Additional Table 4).
[e] $p_D(x)$ indicate frequencies of haplotype x in dams.

Additional Table 4: Calculation of frequency of maternally inherited haplotypes in German Holstein half-sibs with 23del-12del / 23ins-12ins (2/3) diplotype.

Number of Paternally plus Maternally Inherited Haplotypes 23del-12del (2) and 23ins-12ins (3)[a]	Total Frequency of Haplotypes[b]	Approximative Frequency of Maternally Inherited Haplotypes[c]
$n(2) = n(1/2) + 2 \cdot n(2/2) + n(2/3) + n(2/4)$	$p(2) = 0.539$	$p(2)_e = (p(2) - 0.5) \cdot 2 + 0.5$
$n(2) = 634$		$p(2)_e = 0.577$
$n(3) = n(1/3) + n(2/3) + 2 \cdot n(3/3)$	$p(3) = 0.461$	$p(3)_e = (p(3) - 0.5) \cdot 2 + 0.5$
$n(3) = 543$		$p(3)_e = 0.423$

[a] n(x) indicate number of observations of haplotype x.
[b] p(x) indicate frequency of haplotype x.
[c] $p(x)_e$ indicate approximative frequency of maternally inherited haplotype x.

Additional Table 5: Calculation of approximative numbers of diplotypes of German Holstein, assuming Hardy-Weinberg equilibrium.

	Diplotypes					
	1/1	1/2	1/3	2/2	2/3	3/3
	23del-12ins/ 23del-12ins	23del-12ins/ 23del-12del	23del-12ins/ 23ins-12ins	23del-12del/ 23del-12del	23del-12del/ 23ins-12ins	23ins-12ins/ 23ins-12ins
Formula[a,b]	$\frac{n}{2} \cdot (p_D(1))^2$	$\frac{n}{2} \cdot 2p_D(1)p_D(2)$	$\frac{n}{2} \cdot 2p_D(1)p_D(3)$	$\frac{n}{2} \cdot (p_D(2))^2$	$\frac{n}{2} \cdot 2p_D(2)p_D(3)$	$\frac{n}{2} \cdot (p_D(3))^2$
Inferred number of observations	3	30	21	87	123	44

[a] n indicate the total number of diplotype observations (Additional Table 3).
[b] $p_D(x)$ indicate haplotype frequencies as calculated in Additional Table 3 and 4.

1. Stephens M, Donnelly P: **A comparison of bayesian methods for haplotype reconstruction from population genotype data**. *Am J Hum Genet* 2003, **73**:1162-9.
2. Stephens M, Smith NJ, Donnelly P: **A new statistical method for haplotype reconstruction from population data**. *Am J Hum Genet* 2001, **68**:978-89.
